# Prenatal diagnosis of mucopolysaccharidosis type I on hepatosplenomegaly and coarse features: a case-report

**DOI:** 10.1186/s12884-024-07115-5

**Published:** 2025-01-03

**Authors:** Maxime Agranier, Florence Demurger, Christele Dubourg, Jerome Fromageot, Anne-Sophie Cabaret Dufour, Erika Launay, Magalie Gournay, Charles Lefèvre, Roseline Froissart, Magali Pettazzoni, Paul Rollier

**Affiliations:** 1 Laboratory of Cytogenetics and Cell Biology, Rennes University Hospital, Rennes, France; 2 Bretagne Atlantique Hospital, Department of Paediatrics, Vannes, France; 3 Laboratory of Molecular Genetics and Medical Genomics, Rennes University Hospital, Rennes, France; 4 Department of Gynaecology Obstetrics, Bretagne Atlantique Hospital, Vannes, France; 5 Department of Gynaecology Obstetrics, Rennes University Hospital, Rennes, France; 6 Anatomico-Pathological Laboratory, Vannes Hospital, Vannes, France; 7 Laboratory of Biochemistry and Molecular Biology, Lyon University Hospital, Bron, France; 8 Department of Clinical Genetics, Rennes University Hospital, Rennes, France

**Keywords:** Mucopolysaccharidosis I, Prenatal diagnosis, Hepatosplenomegaly, Lysosomal storage diseases, Exome sequencing, *IDUA* gene

## Abstract

**Background:**

Mucopolysaccharidosis type I (MPS I - *IDUA* gene) is a rare autosomal recessive lysosomal storage disorder. Clinical symptoms, including visceral overload, are progressive and typically begin postnatally. Descriptions of hepatosplenomegaly associated with lysosomal pathology are uncommon during the prenatal period. The most prevalent etiologies are infections, anemia, and neoplasms.

**Case presentation:**

A pregnant woman at 26.5 gestational weeks was referred to our center for fetal ultrasound findings of hepatosplenomegaly, distinct facial features, and liver, spleen and thymus echogenic spots. Whole exome sequencing after amniocentesis identified two likely pathogenic *IDUA* gene variants (in *trans*), raising suspicion of a diagnosis of MPS I. MPS I was confirmed by the deficiency of α-L-iduronidase activity in amniotic cells. A medical pregnancy termination was carried out due to the severe prognosis. After termination of pregnancy, external examination of the fetus confirmed hepatosplenomegaly and coarse dysmorphic features.

**Conclusion:**

Lysosomal storage diseases (LSD) are a rare cause of prenatal hepatosplenomegaly, but this has not been described in MPS I according to our literature search. The genetic variants identified in this case prompted early diagnosis through genome-wide studies. This rare presentation of MPS I highlights the expanding role of genomic analyses in diagnosing conditions during pregnancy.

## Background

Mucopolysaccharidosis type I (MPS I) is a rare autosomal recessive lysosomal storage disease (LSD) linked to pathogenic variants in *IDUA* gene. *IDUA* codes for the α-L-iduronidase enzyme and its deficit leads to lysosomal storage of glycosaminoglycans dermatan sulfate and heparan sulfate. Clinical features are variable, ranging from a severe form (Hurler type; OMIM #607014) with onset before 1 year, to milder forms with later onset: Hurler-Scheie (intermediate form; OMIM #607015) and Scheie (mild form; OMIM #607016) types [1]. The incidence of this pathology is estimated at 1 in 100,000 live births for Hurler type to 1 in 800,000 for Scheie type [2]. In the majority of cases of Hurler syndrome, clinical signs appear after birth, and neonatal signs are rare. These clinical signs include musculoskeletal abnormalities (short stature, multiple dysostosis, thoraco-lumbar kyphosis), progressive thickening of facial features (protruding frontal bones, low nasal root with broad tip and anteverted nostrils, round cheeks, thickened lips), cardiomyopathy and valvular anomalies, sensorineural deafness, enlarged tonsils and adenoids. Developmental delay, particularly in speech, typically arises between 12 and 24 months, accompanied by progressive cognitive and sensory decline. Other manifestations include organomegaly, hernia, hirsutism, hydrocephalus, diffuse corneal [3]. The first specific clinical signs only appear after a few months of life, linked to progressive lysosomal overload. MPS I with prenatal visceral presentation is particularly rare. While the combination of hepatosplenomegaly and coarse facial features is highly suggestive of a lysosomal disease in children, these signs have never been reported prenatally in MPS I according to our literature search. Prenatal diagnosis is performed mainly on family history, and a few cases of hydrops have been described, although this is much less frequent than in other lysosomal pathologies [4]. We present what is, to our knowledge, the first case of prenatal MPS I diagnosed based on the presence of antenatal signs of overload, including hepatosplenomegaly and coarse facial features, as early as the second trimester of pregnancy. This diagnosis was confirmed through biochemical and genetic testing.

## Case presentation

A pregnant woman was referred by a partner center at 26.5 gestational weeks (GW) to the prenatal diagnostic center of Rennes (France). This was her second pregnancy following a previous delivery of a healthy infant. The couple was not consanguineous, their phenotype was normal, and they had no significant personal or family histories. Morphologic ultrasound examination conducted during the first trimester revealed a normal nuchal translucency of 2 mm (1.06 Multiple of Median (MoM), Crown-rump length: 77.6 mm) and a single umbilical artery. Additionally, vaginal bleeding related to a placental hematoma was observed.

**Fig. 1:**
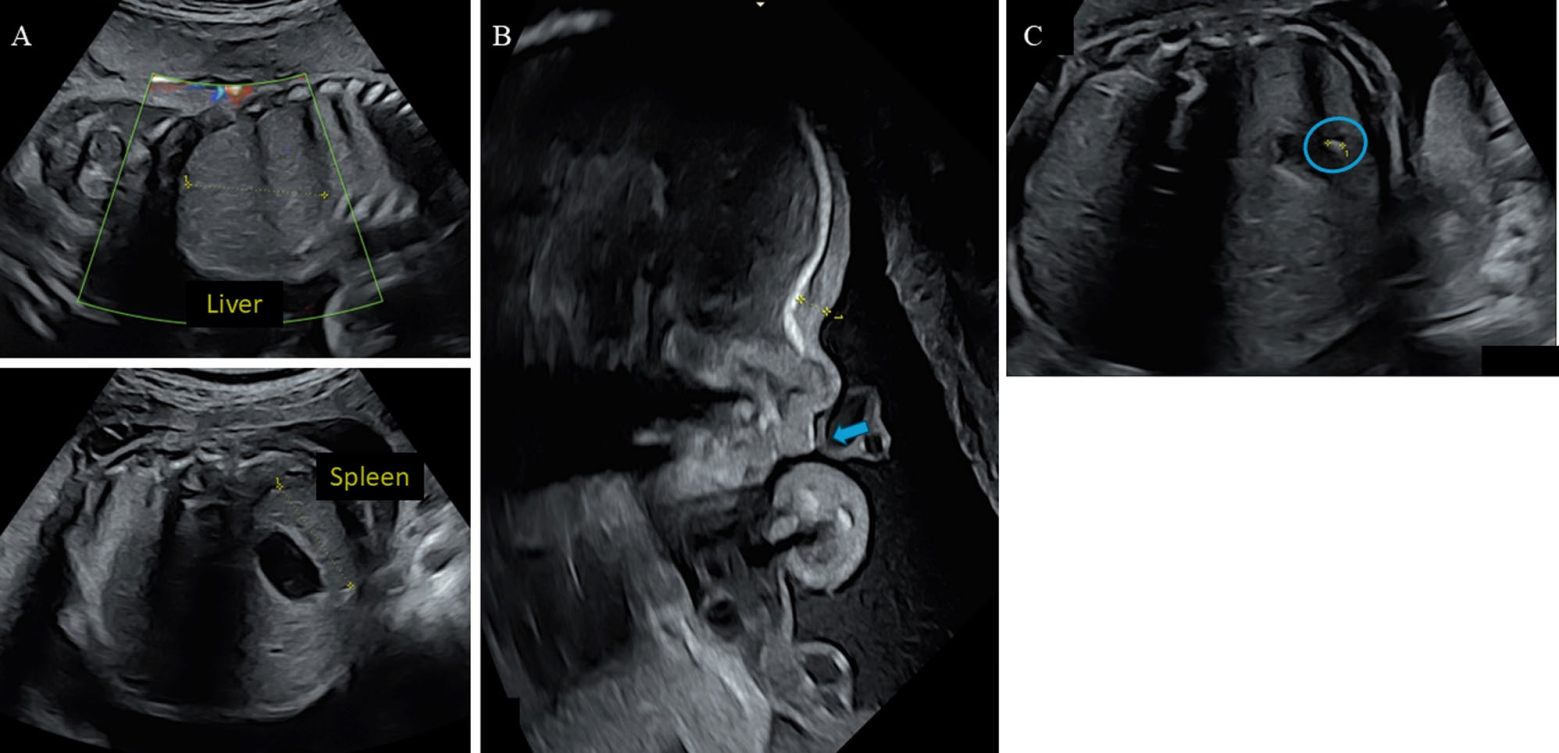
Morphological studies on Fetal Ultrasonic image at 28 GW. **(A)** Hepatosplenomegaly (measurements over + 2 SD). **(B)** Fetal profile with blue arrow pointing to the broad philtrum. **(C)** Peritoneal echogenic punctuation above the stomach (blue circle)

Ultrasound examination at 24.0 GW revealed hepatosplenomegaly (Fig. 1A) and dysmorphic features, including a long and broad philtrum (Fig. 1B), as well as a few echogenic spots in the liver, spleen, peritoneum, and thymus (Fig. 1C). The cytomegalovirus (CMV) profile indicated long-standing immunity. Amniocentesis was performed at 26.7 GW for a chromosomal microarray analysis (CMA) and trio whole-exome sequencing (WES) examination. CMA was normal, but two likely pathogenic variants (class 4 according to ACMG classification) were identified by WES on *IDUA* gene: NM_000203.5:c.[590G > A]; [1139dup]; NP_000194.2:p.[(Gly197Asp)]; [(Leu381Alafs*18)] (Table 1). The presence of these two variants in compound heterozygous state raised suspicion of MPS I. No other variants of interest were identified. MPS I was next confirmed by enzymatic analysis in cultured amniocytes, with evidence of a deficiency in α-L-iduronidase activity (Table 1).

**Table 1:** Biology results: genetic analysis results; α-L-iduronidase enzyme activity in cultured amniocytes

Whole exome and targeted sequencing (fetal DNA)		
Allele 1: NM_000203.5(IDUA): c.[494-57G > A;590 G > A], inherited from the mother		
Allele 2: NM_000203.5(IDUA): c.1139dup, inherited from the father		

α-L-iduronidase enzyme activity in cultured amniocytes.		
	Measured value	Laboratory control
α-L-iduronidase activity	0.3 µkat/kg	33.8 µkat/kg
Hexosaminidase activity (control enzyme)	827 µkat/kg	1687 µkat/kg

**Fig. 2:**
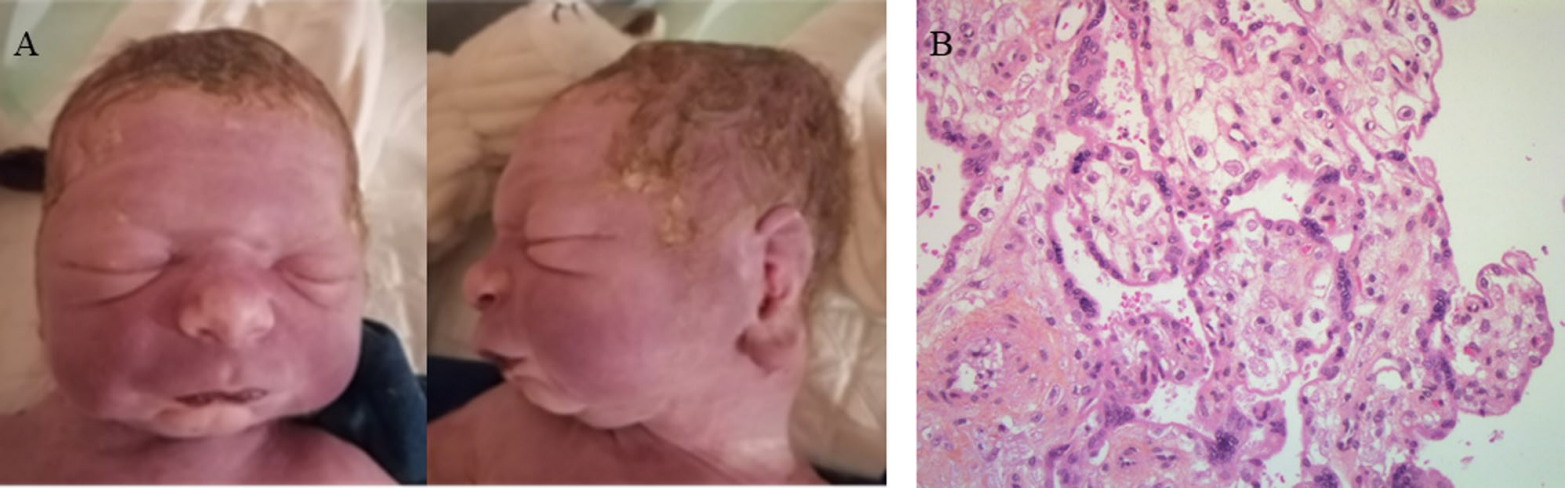
Post-termination studies. **(A)** External examination post-medical abortion at 35 GW; coarse facial features with broad philtrum, broad nasal tip, micrognathia, thin upper lip vermilion, hypertelorism, plagiocephaly, microretrognatism, full and drooping cheeks, bulging or forward-projecting philtrum, large, badly hemmed ears with bulky lobes, bulging eyes, marked suborbital folds. **(B)** Optical microscopic image showing vacuolization of Hofbauer cells (H&E stain; ×100)

The couple elected for a medical termination of pregnancy, which was carried out at 35 GW. In France, pregnancy terminations for medical reasons are permitted until its term when a disease of particular severity is diagnosed in the fetus and is incurable at the time of diagnosis, as is the case for severe MPS I. At the parents’ request, only an external examination was performed. The infant’s birth biometrics were as follows: weight, 3140 g (94th percentile); length, 48 cm (80th percentile); occipitofrontal circumference 34 cm (83rd percentile). External examination confirmed hepatomegaly, with hepatic overhang of 4 cm and dysmorphic features, including coarse facial features, bulging or forward-projecting philtrum, broad nasal tip, micrognathia, thin upper lip vermilion, hypertelorism, plagiocephaly, microretrognatism, full and drooping cheeks, large, badly hemmed ears with bulky lobes, bulging eyes and marked suborbital folds (Fig. 2A). Placenta analysis showed single umbilical artery and micro vacuolized appearance of Hofbauer cells, compatible with lysosomal overload (Fig. 2B).

Given that the substitution variant (c.590G > A) is located at the canonical acceptor site of exon 6, we investigated the possible splicing impact. This was achieved through the use of a Minigene assay (as detailed in Gaildrat et al. [5]). In this construct, the c.590G > A variant is responsible for the appearance of a major transcript with complete retention of intron 5, as well as a few alternative transcripts with retention of the last 22, 25 and 28 nucleotides of intron 5. Complete retention of intron 5 leads to a premature stop codon, p.(Phe198Valfs*127). A second construction, using a longer sequence, revealed the complementary role of a 2nd rare variant (c.494-57G > A), in *cis* of the c.590G > A variant, also altering splicing. This variant creates an additional cryptic splicing site, resulting in the retention of the final 55 nucleotides of intron 4. This, in turn, leads to the formation of a premature stop codon (p.(Arg166Valfs*18)).)). These functional studies (enzyme activity and transcript studies) allowed us to reclassify these variants as pathogenic (class 5 according to ACMG classification).

## Discussion

The etiology of fetal hepatosplenomegaly is multifactorial. It is crucial to determine the underlying cause, as some diagnoses are amenable to treatment or may have subsequent gestational implications (e.g., neonatal hemochromatosis). Major contributors include fetal infections, summarized by the acronym TORCH (Toxoplasmosis, Other infections (Parvovirus, Syphilis, Zika, Chickenpox, HIV), Rubella, Cytomegalovirus, Herpes Simplex). Hepatomegaly may also result from fetal anemia or hepatic tumor, such as hepatoblastoma, hemangiomas, mesenchymal hamartomas… [6]. Among constitutional genetic causes, trisomy 21 (OMIM #190685) is responsible in 5–10% of cases for transient abnormal myelopoiesis [7], a pre-leukemic syndrome which is responsible for hepatomegaly in fetuses and newborns [8]. Wiedemann-Beckwith syndrome (OMIM #130650) combines macroglossia, omphalocele, polyhydramnios, macrosomia and visceromegaly with hepatosplenomegaly [9].

Lysosomal storage diseases (LSD) are a classic yet rare cause of hepatosplenomegaly, with few cases arising during the prenatal period and often associated with others signs like hydrops fetalis and/or fetal ascitis. Indeed, hydrops fetalis is the most frequent presentation indicator of lysosomal pathology, while associated antenatal hepatomegaly is seldom documented. In a context of nonimmune hydrops fetalis, the estimated prevalence of LSD is between 1.3 and 8% [10–12] with the most frequently diagnosed conditions (> 70% of cases) being mucopolysaccharidosis type VII (Sly disease; OMIM #253220), galactosialidosis (OMIM #256540) and sialidosis (OMIM #256550), infantile free sialic acid storage disease (OMIM #269920), Gaucher disease (OMIM #230800), and GM1 gangliosidosis (OMIM #230500). In addition, a significant number of other lysosomal pathologies have been identified at least once as a cause of hydrops fetalis [10, 12], including, but rarely, a few cases of MPS I. Another way LSD may manifest during the prenatal period is chondrodysplasia punctata, as observed in mucolipidosis type II and GM1 gangliosidosis [13], or multiple dysostosis, as in mucolipidosis type II [14].

In MPS I, the earliest signs typically manifest after birth, and are often present from the first month of life but are not necessarily specific: breathing difficulties, otitis media, hearing loss, hernias, hypotonia, feeding difficulties [3]. Consequently, the diagnosis is often made later, except in countries where newborn screening has been introduced [15]. In the MPS I registry study of 115 individuals with Hurler form with no family history, the median age at diagnosis was 0.8 years [2]. The most specific signs are kyphosis, corneal opacity, characteristic coarse facial features and hepatomegaly. However, hepatomegaly is classically one of the later signs, present in 61.4% of patients and detected after a median of 9.8 months in this study [2]. In prenatal care, only a few isolated cases of MPS I with hydrops have been published [16], with most prenatal diagnoses being made because of family history. In France, pregnancy monitoring includes 3 systematic ultrasounds (first 9–11 WG, second 20–22 WG and third trimester 30–32 WG). It is challenging to diagnose lysosomal pathology prior to the second trimester ultrasound, given that the initial ultrasound signs were documented in the literature at this gestational age.

Here, the fetus exhibited indications of visceral overload from the prenatal period. This severe expression of the pathology is consistent with genetic studies that identified two variants resulting in premature stop codons, and therefore probably no mRNA, targeted by non-mediated decay. To date, over 300 pathogenic variants have been described and reported in the *IDUA* gene [17]. These include some over-represented variants (e.g., p.Trp402Ter, p.Gln70Ter, p.Pro533Arg) as well as more complex and difficult to interpret pseudodeficient alleles (e.g., p.His82Gln, p.Ala300Thr) [18]. In severe forms, > 79% of genotypes include at least one nonsense/splice/frameshift variant; however, in many cases (i.e., > 20%), the combination of variants is unique to a single patient [19]. The enzymatic studies corroborate this finding, with a marked decrease in α-L-iduronidase activity. Given the grave ramifications of this disease and the risk of recurrence (25%) for future pregnancies, a prenatal or pre-implantation diagnosis can be offered to the couple.

From a genetic standpoint, it is noteworthy that only the c.590G > A variant of the c.[494-57G > A;590 G > A] complex allele was identified during the WES. The c.494-57G > A variant is located more than 50 bp from the intron-exon junction and was therefore not covered by WES (the presence of this second variant was confirmed by targeted sequencing in the fetus (Table 1)). However, as it is upstream of the c.590 G > A variant, the c.494-57G > A has probably the greatest biological impact, although another substitution (c.589G > A p.(Gly197Ser)) on the same codon as the first variant has already been reported as pathogenic [20, 21]. Without being associated with the c.590 G > A variant, the c.494-57G > A variant alone might not have been detected, and the diagnosis of MPS I might therefore have been delayed or not made at all. In the absence of any previous description of signs of prenatal visceral overload in MPS I, as reported in this case, it is unlikely that the pathology would have been sought by targeted enzymatic techniques, as has been done historically, and as was done here following the genetic suspicion. The advent of prenatal genomics will provide better coverage of these intronic variants and therefore improve diagnostic results [22, 23]. This also underlines the importance of histological analysis of the placenta (and the fetus when feasible) in instances of suspected lysosomal disease, as this can assist in the diagnostic process when a definitive diagnosis has not been reached during the prenatal period. Microscopically, macrophage overload is a constant feature in lysosomal storage disorder, with macrophages being particularly rich in lysosomes. Lysosomal overloading is identified by the presence of vacuoles in affected cells, for example in the placenta, and particularly in Hofbauer cells [24]. Vacuoles may be present as early as the first trimester, though they may only be visible under electron microscopy. In some cases, the location and composition of the vacuoles can assist in formulating a diagnosis [25]. The chorionic villi from the placenta of fetuses with MPS1 displayed a remarkable degree of vacuolation of stromal cells [26], with vacuoles being relatively scarce within the cytotrophoblast and occurred with greater regularity in fibroblasts and endothelial cells [27].

## Conclusion

This case illustrates the growing interest in prenatal studies at the exome or genome level for the diagnosis of rare genetic diseases, making it possible to broaden the clinical spectrum of these diseases, and to make informed decisions for the current pregnancy, in particular when ultrasound signs are not specific, and carrying out a prenatal diagnosis for subsequent pregnancies.

## Data Availability

The datasets used during the current study are available from the corresponding author on reasonable request.All the mentioned variants have been deposited in the ClinVar database and are available under the following references: SUB14978208.

## Abbreviations

ACMG: American College of Medical Genetics and Genomics
CMA: Chromosomal Microarray Analysis
GW: Gestational Weeks
LSD: Lysosomal Storage Disease
MoM: Multiple Of Median
MPS I: Mucopolysaccharidosis type I
WES: Whole-Exome Sequencing
